# Information systems on human resources for health: a global review

**DOI:** 10.1186/1478-4491-10-7

**Published:** 2012-04-30

**Authors:** Patricia L Riley, Alexandra Zuber, Stephen M Vindigni, Neeru Gupta, Andre R Verani, Nadine L Sunderland, Michael Friedman, Pascal Zurn, Chijioke Okoro, Heather Patrick, James Campbell

**Affiliations:** 1 Division of Global HIV/AIDS, Center for Global Health, Centers for Disease Control and Prevention, 1600 Clifton Road, MS-E41, Atlanta, GA 30333, USA; 2 National Center for Environmental Health, Centers for Disease Control and Prevention, Atlanta, GA, USA; 3 University of Washington School of Medicine, Seattle, WA, USA; 4 Department of Human Resources for Health, World Health Organization, Geneva, Switzerland; 5 Instituto de Cooperación Social–Integrare, Barcelona, Spain

**Keywords:** Human resources information system, HRIS, Human resources for health, Literature review, Workforce surveillance, Workforce science, Global health

## Abstract

**Background:**

Although attainment of the health-related Millennium Development Goals relies on countries having adequate numbers of human resources for health (HRH) and their appropriate distribution, global understanding of the systems used to generate information for monitoring HRH stock and flows, known as human resources information systems (HRIS), is minimal. While HRIS are increasingly recognized as integral to health system performance assessment, baseline information regarding their scope and capability around the world has been limited. We conducted a review of the available literature on HRIS implementation processes in order to draw this baseline.

**Methods:**

Our systematic search initially retrieved 11 923 articles in four languages published in peer-reviewed and grey literature. Following the selection of those articles which detailed HRIS implementation processes, reviews of their contents were conducted using two-person teams, each assigned to a national system. A data abstraction tool was developed and used to facilitate objective assessment.

**Results:**

Ninety-five articles with relevant HRIS information were reviewed, mostly from the grey literature, which comprised 84 % of all documents. The articles represented 63 national HRIS and two regionally integrated systems. Whereas a high percentage of countries reported the capability to generate workforce supply and deployment data, few systems were documented as being used for HRH planning and decision-making. Of the systems examined, only 23 % explicitly stated they collect data on workforce attrition. The majority of countries experiencing crisis levels of HRH shortages (56 %) did not report data on health worker qualifications or professional credentialing as part of their HRIS.

**Conclusion:**

Although HRIS are critical for evidence-based human resource policy and practice, there is a dearth of information about these systems, including their current capabilities. The absence of standardized HRIS profiles (including documented processes for data collection, management, and use) limits understanding of the availability and quality of information that can be used to support effective and efficient HRH strategies and investments at the national, regional, and global levels.

## Background

In 2000, the global community made a historic commitment to combat poverty and its main causes and manifestations by agreeing to eight Millennium Development Goals (MDGs), four of which directly target improved population health outcomes through strengthening of health systems and human resources for health (HRH) [1]. The recognition that attainment of the health-related MDGs is directly related to an adequate supply and distribution of trained health workers has resulted in advocacy for increased action and investment in HRH [2-6], especially among 57 resource-limited countries identified by the World Health Organization (WHO) as experiencing acute workforce shortages [2]. This awareness has been accompanied by international calls to strengthen “systems for recording and updating health worker numbers [that] often do not exist, which presents a major obstacle to developing evidence-based policies on human resources development,” such as articulated in the WHO’s *World Health Report 2006*[2]. Table 1 identifies eight recent global and regional calls to action for strengthening the information and evidence base to support HRH policy and planning [7-14].

**Table 1:** International calls to action for strengthening the HRH information and evidence base in countries, 2006-2011

**Global and regional commitments**	**Relevant text related to strengthening HRH information, evidence and monitoring**
**1. Outcome Statement of the Second Global Forum on Human Resources for Health** (2011)	“There is a need for strong national capacity in all countries to regularly collect, collate, analyze and share data to inform policymaking, planning, and management… Attention should be paid to aspects such as geographic distribution, retention, gender balance, minimum standards, competency frameworks, and reflect the diverse composition of the health workforce.”
2. World Health Assembly (WHA) Resolution 63.16: **Global Code of Practice on the International Recruitment of Health Personnel** (2010)	Member States should:
	· establish or strengthen and maintain, as appropriate, health personnel information systems, including health personnel migration, and its impact on health systems.
	· collect, analyse and translate data into effective health workforce policies and planning.
**3. Kampala Declaration and Agenda for Global Action on HRH**. Adopted at the First Global Forum on Human Resources for Health (2008)	Calls upon countries:
	· to create health workforce information systems, to improve research and to develop capacity for data management in order to institutionalize evidence-based decision making and enhance shared learning.
	· to develop standardized indicators and strengthen statistical capacity… [and to] monitor health workforce flows in and out of countries, making such data transparently available and using this information to inform policy and management decisions.
	“Improved information, data and research… will be the basis for accountability between partners, stakeholders, countries and regions.”
4. WHA Resolution 60.27: **Strengthening Health Information Systems** (2007)	Urges Member States:
	· to mobilize the necessary scientific, technical, social, political, human and financial resources in order [to] establish and operationalize health information systems as a core strategy for strengthening their national health systems.
	· to determine programme-based information systems as subsets of national health information systems [and] to organize the harmonization of the various programme(s).
5. African Union: **High Level Inter-ministerial Technical Consultation on Strengthening Political Support for Health Worker Development in Africa** (2007)	“The quality of information on the health workforce available at national and regional levels should be improved. This requires investment at the national level in both research to identify health workforce needs and motivation and improving data collection for monitoring health worker numbers, distribution and mobility…”
6. Pan American Health Organization Resolution CE140.R13: **Regional Plan of Action for Human Resources for Health** (2007)	Urges Member States:
	· to consider developing a national plan of action for human resources for health, with specific goals and objectives, an appropriate set of indicators and a tracking system.
7. European Commission: **European Programme for Action to Tackle the Critical Shortage of Health Workers in Developing Countries** (2006)	“The EU will support the mapping [and] analysis… on human resources necessary for effective advocacy and action… The EU will support [mechanisms to] collect, collate and analyse data, and disseminate information and advocate policy based on national HR information.”
8. WHA Resolution 59.27: **Strengthening Nursing and Midwifery** (2006)	Urges Member States:
	· to provide support for the collection and use of nursing and midwifery core data as part of national health-information systems.

Source: [7-14].

While there is no single data source or indicator that can capture the various dynamics of HRH stocks and flows, a number of different data sources exist in most countries that can potentially be used to glean information about the health workforce, including population censuses, labor force and employment surveys, health facility assessments, and administrative databases for human resources management (e.g. health professional licensing and payroll databases) [15,16]. However, many of these sources were not designed for the specific purpose of supporting HRH policy and planning. The global evidence-base regarding the impact of HRH on health systems and health outcomes remains largely fragmented and incomplete, partly due to a lack of institutional capacity in many countries to collect and use these data to support HRH decisions, and partly due to a lack of awareness among the international community [5,15]. Until recently, tools for measuring and monitoring HRH offered few norms and standards for comparison, and available data are still largely inconsistent within and between countries, which limits opportunities for understanding effective workforce strategies and interventions [15-19].

Increasingly, systems for collecting and disseminating information on a country’s health workforce – referred to as human resources information systems (HRIS) – are becoming an integral component to national HRH performance assessment and systems strengthening frameworks [15,20-24]. Functional HRIS models involve standardized processes for data capture, management, and use so as to provide accurate, timely, and comprehensive profiles of workforce size, composition, and deployment [15,25-27]. When comprehensively designed and implemented, HRIS empower decision makers to anticipate a variety of HRH issues, such as an insufficient supply of younger workers entering the health system who can offset employee departures due to retirement, death, or out-migration. They facilitate meaningful integration of workforce data across multiple information points, for example, by ensuring health ministries (and other employers) that health professionals on staffing or payroll records are appropriately credentialed and qualified to practice (based on registries of professional regulatory bodies). They are used to collate data on workers in government-operated health facilities, private (for-profit or non-profit) and parastatal facilities, as well as those working outside of facility-based service delivery. The systems collect data on all human resources: physicians, nurses, and midwives, plus other categories of allied health professionals and technicians, as well as the management and support personnel necessary for sound health system functioning. In addition, linking HRIS data with broader health information – such as disease burden, health services utilization, and patient outcomes – can be a powerful tool in prioritizing resource allocation for health worker training and deployment in order to meet health system goals.

The need for quality information is pressing for ensuring greater efficiencies in health systems, as well as for ensuring improved accountability and good governance through performance monitoring of national and donor-supported HRH initiatives [18,23,27]. Because countries with varying stages of social and economic development are at different stages in HRIS development and use, there is a heightened need for global consensus regarding standardized approaches to assessing HRIS performance.

In 2010, with five years remaining to the MDGs deadline, stakeholders representing WHO, Health Metrics Network (HMN), Global Health Workforce Alliance, and other national and international agencies convened the Health Workforce Information Reference Group (HIRG), a technical working group focused on prioritizing national HRH data collection and use, including building capacity in HRIS strengthening and assessment [28]. While the HIRG has noted that tools to support HRIS development are growing [15,29-31], there was also collective recognition that scant baseline information existed regarding the scope and adequacy of existing HRIS implementation worldwide.

Since there has not been an extensive study of HRIS implementation on a global scale thus far, we conducted a comprehensive literature review in order to draw a baseline portrait. The objectives of the review were to: (i) review and systematically assess national practices in HRIS implementation worldwide; (ii) identify the main areas of weakness in HRIS implementation, with attention to countries facing acute health workforce shortages; and (iii) draw upon documented best practices to offer recommendations to ministries of health and global health policy makers on how to improve the science and application of human resources information and monitoring systems.

## Methods

Our study entailed a structured review process of scientific publications and grey literature. Given the innovative nature of our research, we adapted the Cochrane methodology of conducting systematic reviews on the effects of health care interventions [32]. Following searches of several global and regional bibliographic databases, we employed a more flexible search strategy to better capture unpublished and grey literature. We also developed a unique quantitative data abstraction tool appropriate to the topic.

### Publications in scientific (peer-reviewed) journals

A broad systematic literature search was conducted in four major electronic databases: MEDLINE (including in-process and non-indexed citations), Excerpta Medica database (EMBASE), PsycInfo, and Cumulative Index to Nursing and Allied Health Literature (CINAHL), using the search concepts “health personnel,” “information system,” “HRIS,” and “health care worker and tracking” (see Table 2)*.* Database searches captured multiple languages (English, French, Portuguese, and Spanish articles) and covered the period 1959–2009.

**Table 2:** Search strategy for HRIS literature in bibliographic databases

**General search term**	**Additional search terms**
***Health personnel***	(health and personnel or health manpower or workforce or human resource or labor market), or health care worker or nurse or physician or midwife or midwives or laboratory technologist or laboratory technician or laboratory worker or laboratory professional or lab technologist or lab technician or lab worker or lab professional or pharmacist or health worker or clinical officer
***Information system***	tracking or informatic or distribution or database or (labor or work or human resource or employ or personnel or staff), (system or program or data or surveillance) or (labor or work or human resource or employ or personnel or staff) or geographic information systems or resource allocation or workplace or databases, factual or Public Health Informatics or Health Care Rationing or “Personnel Staffing and Scheduling” or (tracking or informatic or distribution or database or information system) or (labor or work or human resource or employ or personnel or staff), (system or program or data or surveillance or supply or vacant)
***HRIS***	workforce tracking system or HRIS or human resource information system or human resources survey or human resource cohort studies or workforce surveillance system or labor market survey or workforce capacity or human resources surveillance or (health facility surveys and human resource) or (regulatory board data and employer data)
***Health care worker and tracking***	(health care worker or health worker or nursing or nurse or physician or midwife or midwives or laboratory technician or laboratory worker or laboratory professional or lab technologist or lab technician or lab worker or lab professional or pharmacist or clinical officer) (tracking or informatic or distribution or database or information system or supply)

Additional searches were conducted in the following bibliographic databases:

· LILACS (Literatura Latino-Americana e do Caribe em Ciências da Saúde)

· Global Health

· Sociological Abstracts

· Social Service Abstracts

· ERIC (Education Resources Information Center)

· Web of Science (SCI and SSCI)

· Cochrane.

Two different reviewers assessed the titles and abstracts of articles retrieved through the searches to determine relevancy to the specific objectives of this study. Exclusion criteria included articles on information systems that did not pertain to the health workforce, or that cited terms such as “HRIS” but did not describe a national HRIS implementation process in sufficient detail to be reviewed.

### Grey literature

A review of grey literature was also conducted and encompassed 26 global, regional, and national databases and websites, mostly related to HRH and health information (Table 3). Given the frequency of grey literature updates on the topic, we limited our review to reports dated within the last decade (1999–2010). Search terms were modified to reflect more policy-relevant terminology and included: “human resource for health information system,” “HRH profile,” and “human resource information system”. Additional literature identified through application of a snowball search methodology included book chapters, working papers, conference proceedings, government reports, assessments supported by development agencies, and dissertations. Excluded were PowerPoint presentations, marketing or promotional documents, and reports or databases with HRH statistics that did not include sufficient metadata on the nature and processes of the data collection and management systems within countries used to generate summary statistics. Duplicate articles (e.g. available in different languages or from different websites) were identified and counted once.

**Table 3:** Databases and websites searched for grey literature on HRIS

**Organization or database name**	**URL**
**Africa Health Workforce Observatory**	http://www.hrh-observatory.afro.who.int/
**Americas Regional Observatory of Human Resources in Health**	http://www.observatoriorh.org
· *Basic data*	http://www.observatoriorh.org/eng/basic_data.ht
**Asia-Pacific Action Alliance on Human Resources for Health**	http://www.aaahrh.org/
**Canadian Health Services Research Foundation**	http://www.chsrf.ca
**Canadian Institute for Health Information**	http://www.cihi.ca
· *Spending and health workforce*	http://www.cihi.ca/CIHI-ext-portal/internet/EN/Theme/spending+and+health+workforce/cihi010658
**Capacity Project: HRH Global Resource Center**	http://www.hrhresourcecenter.org
· *Health information systems*	http://www.hrhresourcecenter.org/taxonomy/term/87
**Eastern Mediterranean Regional Observatory on Human Resources for Health**	http://www.emro.who.int/hrh-obs/hrh_about.htm
**European Observatory on Health Systems and Policies**	http://www.euro.who.int/observatory
**Global Health Workforce Alliance**	http://www.who.int/workforcealliance/en/
**Google and Google Scholar**	http://www.google.com
**Health Metrics Network**	http://www.whoint/healthmetrics/support
**Health Systems 20/20**	http://healthsystems2020.org/
**Institute of Development Studies: Eldis**	http://www.eldis.org/
**International Centre for Human Resources in Nursing**	http://www.ichrn.org/
**International Council of Nurses: Innovations Database**	http://www.icn.ch/innovations
**Ministry of Health of Brazil**	http://bvsms.saude.gov.br
· *Chamber of Work Regulation in Health*	http://bvsms.saude.gov.br/bvs/publicacoes/cart_camara_regulacao.pdf
**Ministry of Health of the Republic of Columbia**	http://www.consultorsalud.com/
· *Index of documents*	http://www.consultorsalud.com/biblioteca/documentos/
**Ministry of Health of Mexico**	http://www.mex.ops-oms.org/
**Ministry of Health of Mozambique**	http://www.misau.gov
**Pan American Health Organization**	http://new.paho.org/
**Public Health Informatics Institute**	http://www.phii.org/
**Routine Health Information Network**	http://rhinonet.org/
**United States Department of Health and Human Services: Health Workforce Studies**	http://bhpr.hrsa.gov/healthworkforce/default.htm
**World Health Organization: Health Workforce Statistics**	http://www.who.int/hrh/statistics/en/
**World Health Organization European Regional Office: Health Systems**	http://www.euro.who.int/en/what-we-do/health-topics/Health-systems
**World Health Organization South-East Asian Regional Office: Human Resources for Health**	http://www.searo.who.int/EN/Section1243/Section1308.htm

### Review process

**Figure 1:**
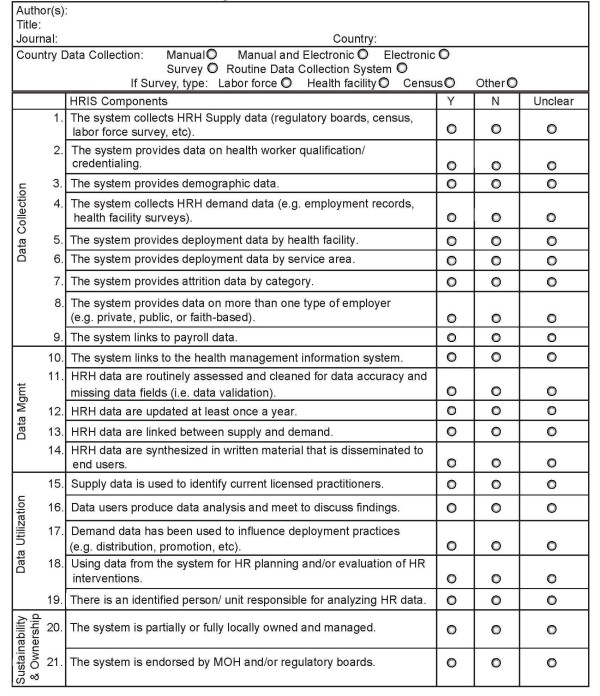
HRIS abstraction tool.

Articles that met the initial selection criteria were assigned to a review pair (two individuals) for full review and analysis. So as to provide an objective assessment of the HRIS literature, the review team developed an abstraction tool (Figure 1) including elements seen as critical for an effective HRIS. Twenty-one HRIS performance descriptors were grouped into four categories: (i) data collection, (ii) data management, (iii) data utilization, and (iv) system sustainability and ownership. Different indicators of HRIS performance were recorded in each category as to whether or not the process was in place, with a default “unclear” option for ambiguous or omitted descriptions. Instructions and definitions accompanied the tool so as to ensure consistency of abstraction across reviewers and across types of articles retrieved.

The “data collection” category (items 1–9) assesses a system’s ability to quantify the active workforce, including the supply (or stock) of health workers as registered with professional regulatory boards or captured in a population census or labor force survey, and whether it collected information on their deployment (i.e. labor force absorption data as captured through employment records or health facility surveys), distribution (information on numbers of health workers across different sectors), and the professional skill mix (numbers across different occupational groups). This category also documents the availability of information on dimensions of workforce entry, notably those in pre-service training institutions (e.g. medical and nursing schools), as well as on workforce exit (attrition). The “data management” category (items 10–14) documents the presence (or absence) of safeguards to ensure HRH data were cleaned, validated, updated, and rendered usable for analysis and dissemination. The “data utilization” category (items 15–19) assesses HRIS usage for workforce planning and decision-making; and the fourth category, “sustainability and ownership” (items 20–21), captures information which would indicate whether the system being described was locally owned and endorsed.

At least two reviewers read and scored each article using the abstraction tool. Assignment was based in part on the reviewers’ different language skills. When two or more articles were associated with a single country, they were assigned to the same review team. Articles describing HRIS processes across countries within a region were assigned to the review team covering the most single countries within that region. For countries with multiple articles, a composite score for each item captured as “yes” if the process was identified in any of the articles as being in place. Reviewers were not assigned manuscripts on which they were listed as authors or self-identified as peer reviewers prior to publication. Following the individual reviews, each review pair completed a joint abstraction record per article/country, which reconciled any differences and represented the pair’s collective assessment. All abstraction results were entered into *EpiInfo* version 3·5·1 [33] with a linked Microsoft Access database.

### Analysis

Country-specific results on documented HRIS implementation were stratified as to whether countries were designated by WHO as experiencing crisis levels of HRH shortage (“crisis country”) or not (“non-crisis country”). The specific definition is whether the country met a minimum threshold of 23 physicians, nurses, and midwives per 10 000 population needed for the provision of essential health services to meet the MDGs [2]. Frequency analysis was conducted for each grouping, using Microsoft Excel for graphic display.

## Results

### Literature review of national practices in HRIS implementation

**Figure 2:**
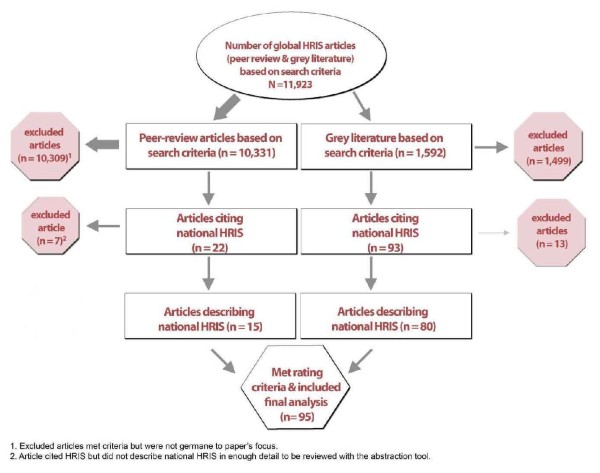
Flowchart of selection process for reviewed articles.

**Figure 3:**
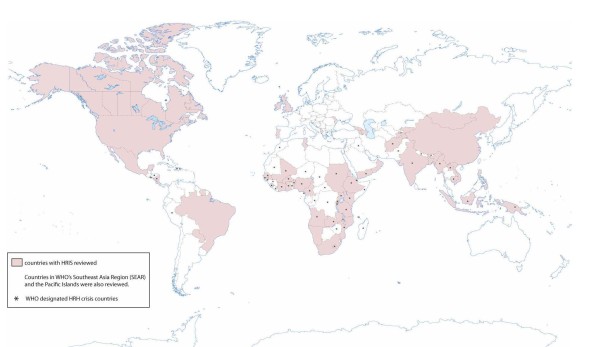
Country human resource information systems (HRIS) reviewed.

Figure 2 presents a flowchart describing the process used to select relevant articles from both the published and grey literature. Based on our broad search criteria, a total of 11 923 articles were initially identified. After exclusion, 95 relevant articles on HRIS performance remained, representing 63 countries: 32 crisis countries (over half of the 57 total) and 31 non-crisis countries. Two systems were documented from a regional perspective, representing Southeast Asia/Pacific Islands and Mercosur. Figure 3 displays the countries and regions for which documentation was found.

Eighty (84 %) of the 95 reviewed articles were from the grey literature and represented the majority of findings. National adaptations of the WHO/HMN’s *Framework and Standards for Country Health Information Systems* and related tools [29,30] provided a significant source of documentation (54 % of the grey literature and 45 % of all articles combined). The full list of reviewed articles is available in an additional file 1. None of the articles dated prior to the year 2000.

### Analysis of strengths and weaknesses in HRIS implementation processes

**Figure 4:**
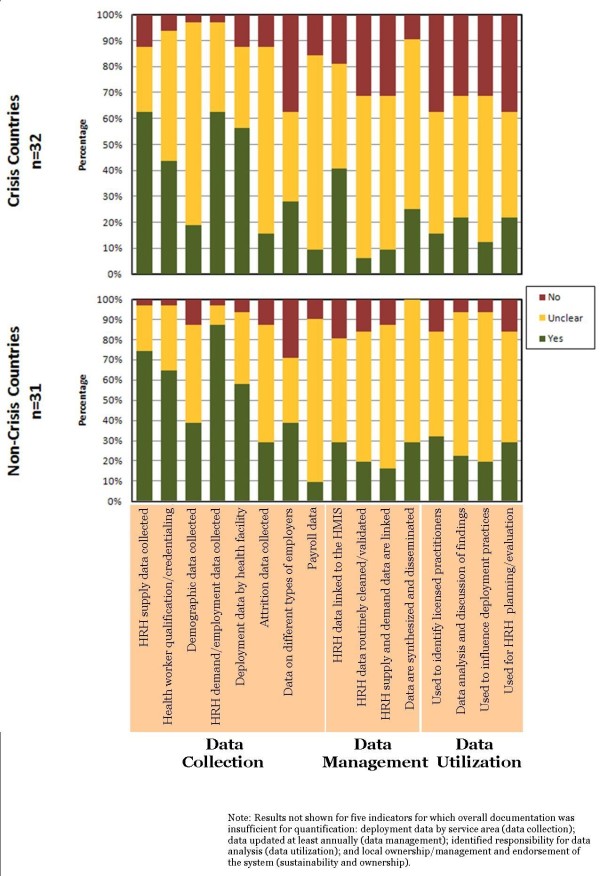
**Documented components of HRIS performance in “crisis” and non-crisis countries (63 countries).** Note: Results not shown for five indicators for which overall documentation was insufficient for quantification: deployment data by service area (data collection); data updated at least annually (data management); identified responsibility for data analysis (data utilization); and local ownership/management and endorsement of the system (sustainability and ownership).

Figure 4 presents the results of the national HRIS performance ratings stratified by crisis and non-crisis countries. Significantly, “unclear” documentation--reflecting an ambiguous or omitted description of a key HRIS feature and illustrated by the yellow bar--was the most common rating for 11 indicators, and this held true for both crisis and non-crisis countries. For five of the original 21 indicators (see Figure 4), overall documentation was insufficient for quantification and so these results are not presented here, including the two indicators comprising the category “Sustainability and Ownership”. Additionally, many of the articles published with donor support included references to local agencies. Some inserted the health ministry’s logo on the report cover without describing how the system was locally sustained or nationally owned. Since explicit description of sustainability and ownership was a prerequisite for quantification using our study approach and tool (refer to Figure 4, indicator 20), these documents were not counted as evidence of local HRIS ownership and management.

In terms of data collection, a high percentage of both crisis and non-crisis countries had documentation on their workforce supply data collection processes, 63 % and 74 %, respectively. Workforce deployment data were also frequently documented in crisis (63 %) and especially non-crisis countries (87 %). Of all systems examined, however, only 23 % reported explicitly collecting data on workforce attrition – information that is especially relevant in countries experiencing HRH rural exodus, international out-migration, or both. Additionally, only a minority (44 %) of HRH crisis countries explicitly reported their HRIS collected data on health worker qualifications or professional credentialing. Few country systems (19 or 29 %) reported collecting data on the demographics of their health workforce (e.g. age, gender, marital status) with fewer crisis countries (19 %) collecting these data compared to non-crisis countries (39 %). One-third (34 %) of the country systems we reviewed collected HRH data from more than one type of employer (e.g. public sector, private for-profit sector, private non-profit sector or faith-based facilities) – 28 % of crisis countries compared to 39 % of non-crisis countries.

With regard to data management, documented linkages across different types of potential HRH and health data sources were limited: 14 % of national/regional HRIS had documented linkages between HRH supply and deployment data, 11 % linked payroll to other types of HRH data, and 34 % linked the data contained within the HRIS to an overall health management information system (HMIS). The latter figure may have been skewed upward by the dominance of literature based on the WHO/HMN tool designed for general health information. Few (6 %) of HRH crisis countries documented their processes and capability for data cleaning and quality control, compared to 19 % of non-crisis countries.

Lastly, in the category of data utilization, few (16 %) of HRH crisis countries documented the use of their HRIS data for identifying licensed practitioners in the labor market. In contrast, roughly twice as many (32 %) of non-crisis countries reported this capability. For crisis and non-crisis countries, the majority of HRIS reports did not indicate whether HRH data actually influenced HRH policy and planning.

Among high-income countries, the Canadian and the United States’ systems were among the better described in terms of having the key features of an effective HRIS. Brazil’s system was the best documented among middle-income countries. The national systems of Kenya and Malawi (both HRH crisis countries) as well as Swaziland stood out as especially promising among low-income countries (results not shown).

## Discussion and conclusions

Despite growing demand at the national and international levels for improved HRH data and analysis to support evidence-based policy, planning, and programming, our findings highlight the critical gap regarding HRIS processes capable of generating needed information. Our review demonstrates the dearth of publicly available information on HRIS implementation and the limited ability to identify criterion for standard practices regarding nationally generated data that are essential for sound HRH decision-making. While there is scant HRIS documentation in the grey literature, HRIS scientific literature published in peer–reviewed journals are even rarer.

Among the 63 documented national HRIS experiences, the literature reviewed could only confirm that while crisis and non-crisis countries tend to generate basic HRH supply and deployment data, few seem to be explicitly using this information for making workforce decisions. In particular, countries with acutely scarce resources, those most in need of efficient HRH utilization, frequently lacked systematic capability to collect or retrieve information on different dimensions of workforce dynamics, such as qualifications, distribution, and retention. Few HRIS were documented as collecting workforce demographic data (e.g. age and sex), essential for effective HRH planning. The small number of countries reported to be collecting data on workforce attrition further underscores the limited global capacity to monitor implementation of the WHO’s recently adopted Global Code of Practice on the International Recruitment of Health Personnel [8]. As noted in Table 1, this new Code also advocates for establishing or strengthening national HRIS especially with regard to health personnel migration.

Complicating any understanding of HRIS performance is the lack of documented ability of most information systems to effectively capture data on different types of health workers from more than one type of employer and link them across service areas. When juxtaposed to the major global and regional commitments to supporting HRH development in resource-limited countries, this imbalance underscores the importance for addressing the paucity of available HRH information and the need for more focus on HRIS strengthening and reporting. The fragmentation of data generation, management, and use found in this review indicates that, of the currently documented data systems, few are capable of drawing a strategic and dynamic picture of the health workforce and its ability to meet population needs.

As previously noted, this review only assessed information that had been documented in selected languages in peer-reviewed journals or grey literature. Some areas of the world may not have been well captured, notably much of the Eastern Mediterranean and European regions. So as to ensure uniformity of data abstraction, articles with insufficient information regarding HRIS implementation were excluded. Our searches did not include specific concepts that might have better identified documents about national HMIS models in which HRH data were systematically embedded. As a result, these findings may have overlooked promising HRIS models that have yet to be publicly described. Also, some HRIS performance indicators originally included in our abstraction tool, notably those regarding system sustainability and ownership, were excluded from the final results due to lack of meaningfully extracted information; this dimension may not be relevant to all countries across levels of development. Since, to the best of our knowledge, standardized abstraction tools specific for HRIS assessment did not previously exist, this review required the creation of an instrument to facilitate data retrieval, one that was not pre-tested or adapted from validated instruments. More formal validity testing of our tool is needed to strengthen its scientific application.

Despite these limitations, we believe this study provides much needed information on the current global status of HRIS performance, one which can serve as both a catalyst for further research generation and dissemination on this topic, as well as a baseline for future investigations. For one, our searches of bibliographic databases uncovered no articles dated prior to the year 2000, highlighting the innovative nature of this topic.

Based on this literature review, our findings indicate the need for more attention to strengthening and reporting the following HRIS features:

· Capability to collect and collate data on HRH across the working lifespan and from multiple sources including payroll records, professional regulatory bodies (where applicable), pre-service training institutions, and population-based census and survey sources;

· Capability to collect and collate deployment data across multiple sectors (e.g. public, private-for-profit, private non-profit, faith-based);

· Capability to link HRH data to broader health information, such as data on population needs, service utilization, and patient outcomes;

· Implementation of methods for data cleaning, validation, and management that allow regular updates (e.g. at least once per year for administrative data); and

· Use of HRH data for policy and practice including strategies for the training, deployment, and retention of health workers.

The scarce documentation of HRIS reflects the current deficiencies in the availability of comprehensive HRH information for use by governments and other stakeholders. Despite increased focus on the inadequate supply and distribution of health workers in low-resource countries − and increasingly in developed countries − support for the development and evaluation of systems to generate the critical HRH data remains limited. Further research on HRIS implementation processes is needed to guide efficient and effective approaches to inform and evaluate health workforce policies and investments at the national and global levels.

## Conflict of interest and funding

We declare that we have no conflicts of interest and that there was no institutional or organizational funding awarded for this study.

## Author contributions

PR, JC, and AZ conceptualized the study and designed the protocol. PR, AZ, AV, and NG conducted the literature search. SV and NS conducted data entry and analysis. PR, AZ, AV, and JC prepared the first draft of the manuscript. All authors participated in the literature review, contributed to the interpretation of the results, and read and approved the final version.

## Supplementary Material

Additional file 1 Reviewed articles on HRIS implementation processes (peer reviewed and grey literature). Click here for file
