# Supplementary material for: Information systems on human resources for health: a global review
Source: Hum Resour Health. 2012 Apr 30;10:7. doi: 10.1186/1478-4491-10-7 (PMC3433380; doi:10.1186/1478-4491-10-7)
Supplement: Additional file 1 — Reviewed articles on HRIS implementation processes (peer reviewed and grey literature). [file 1478-4491-10-7-S1.pdf]

Reviewed articles on HRIS implementation processes (peer reviewed and grey literature)

Article no.	Country no.	Country	Title	Author/Publisher	Source	Year
1	1	Afghanistan	*Afghanistan Health Information System: Review and Assessment*	WHO Health Metrics Network	http://www.who.int/healthmetrics/en/	2007
2	2	Albania	*Assessment of the health information system in Albania*	WHO Health Metrics Network	http://www.who.int/healthmetrics/en/	2008
3	3	Angola	*Angola Health System Assessment*	Connor C, Rajkotia Y, Lin Ya-Shin, et al. Partners for Health Reform.	www.healthsystems2020.org/files/1672_file_Tech079_fin.pdf	2005
4	3	Angola	*Evaluation on the use and sources of HRH data in Angola*	European Commission	European Commission. PALOP Initiative (African Countries of Portuguese Official Language)	2009
5	4	Armenia	*Assessment report health information system of the Republic of Armenia*	WHO Health Metrics Network	http://www.who.int/healthmetrics/en/	2007
6	5	Azerbaijan	*Assessment of National Health Information System in Azerbaijan*	WHO Health Metrics Network	http://www.who.int/healthmetrics/en/	2008
7	6	Benin	*Evaluation du systeme national d'information et de gestion sanitaires (SNIOS) du Benin*	WHO Health Metrics Network	http://www.who.int/healthmetrics/en/	2007
8	7	Brazil	*Use of administrative data sources for health workforce analysis*	McQuide P, Settle D, Abubaker W, et al	WHO *Handbook on Monitoring and Evaluation of Human Resources for Health: With Special Applications for Low and Middle Income Countries* , http://whqlibdoc.who.int/publications/2009/9789241547703_eng.pdf	2009
9	7	Brazil	*Brazil Ministry of Health - descriptions of CONRPOF (professional councils database) and SiGTRABALHO (health workforce database)*	Brazil Ministry of Health	http://portal2.saude.gov.br/portal/saude/profissional/area.cfm?id_area=1293	n.d.
10	7	Brazil	*A informacao para a gestao local de recursos humanos da saude: o sistema de informacao e gestao de recursos humans em saude (SIG-RHS)*	Dal Poz, MR	http://www.obsnetims.org.br/adm/arq/artigo/346063.PDF	n.d.
11	7	Brazil	*Avaliacao de processo na implementacao de politicas publicas: a implantacao do sistema de informacao e gestao humans em saude (SIG-RHS) no contexto das reformas sectoriais.*	Pierantoni CR, Vianna AL, Faculdade de Medicina da Universidade de São Paulo	*Physis: Revista de Saúde Coletiva* 2003: 13 no.1 Rio de Janeiro Jan./June 2003; http://www.scielo.br/scielo.php?pid=S0103-73312003000100004&script=sci_arttext	2003
12	7	Brazil	*Brazil Ministry of Health - Report on Mercosur for Health Work (Annex I : data collection form for health worker database)*	Brazil Ministry of Health	http://portal2.saude.gov.br/portal/arquivos/pdf/cartmercosulespanhol.pdf	2006
13	8	Burkina Faso	*Reseau de metrologie sanitaire d'evaluation du systeme d'information sanitaire*	WHO Health Metrics Network	http://www.who.int/healthmetrics/en/	2008
14	9	Burundi	*Rapport d'évaluation du Système d'Information Sanitaire à l'aide de l'outil HMN*	WHO Health Metrics Network	http://www.who.int/healthmetrics/en/	2008
15	10	Cambodia	*Cambodia Health Information System: Review and assessment*	WHO Health Metrics Network	http://www.who.int/healthmetrics/en/	2007
16	11	Cameroon	*Analyse situationnelle du systeme d'information au Cameroun*	WHO Health Metrics Network	http://www.who.int/healthmetrics/en/	2007
17	12	Canada	*Spending and health workforce*	Canadian Institute for Health Information	http://secure.cihi.ca/cihiweb/dispPage.jsp?cw_page=hhrdata_e	2010
18	13	China	*Health Metrics Network, China Health Information System Review and Assessment*	WHO Health Metrics Network	http://www.who.int/healthmetrics/en/	2006
19	13	China	*China's Human resources for health: quantity, quality and distribution*	Anand S , Fan VY, Zhang J, Zhang L, Ke Y, Dong Z, Chen LC	*Lancet* 2008; 372: 1774-1781 http://www.thelancet.com/journals/lancet/article/PIIS0140-6736(08)61363-X/abstract	2008
20	14	Costa Rica	*Basic Data on Human Resources in the Health Sector in Costa Rica*	Observatory of Human Resources in the Health Sector Reforms, PAHO, WHO	www.observatoriorh.org/eng/basic_data.html	2000
21	14	Costa Rica	*Costa Rica Health System Profile*	PAHO, WHO	http://www.lachealthsys.org/index.php?option=com_docman&task=cat_view&gid=63&Itemid=99999999	2002
22	15	Cote d'Ivoire	*Comprehensive Assessment of Human Resources for Health in Côte d'Ivoire*	Butera D, Fieno JV, Diarra SD, Kombe G, Decker C, Oulai S, Partners for Health Reform Plus, USAID	http://www.hrhresourcecenter.org/node/543	2005
23	15	Cote d'Ivoire	*Rapport d'Evaluation du Systeme National d'Information Sanitaire Ivoirien par l'outil du Resau de Metrologie Sanitaire RMS/HMN*	WHO Health Metics Network	http://www.who.int/healthmetrics/en/	2009
24	16	Dominican Republic	*Dominican Republic Health System Profile*	PAHO, WHO and USAID	http://www.lachealthsys.org/index.php?option=com_docman&task=cat_view&gid=63&Itemid=99999999	2007
25	16	Dominican Republic	*Report for the Observatory of Human Resources in Health in the Dominican Republic*	PAHO, WHO	www.observatoriorh.org/eng/basic_data.html	2001
26	17	El Salvador	*Compilation of Basic Data about Human Resources for Health*	WHO Health Metrics Network	http://www.who.int/healthmetrics/en/	2008

Article no.	Country no.	Country	Title	Author/Publisher	Source	Year
27	18	Eritrea	*Eritrea Health Information System Assessment Report*	WHO Health Metrics Network	http://www.who.int/healthmetrics/en/	2007
28	19	Ethiopia	*Country Case Study: Ethiopia's Human Resources for Health Program*	Global Health Workforce Alliance, WHO	http://www.who.int/workforcealliance/knowledge/case_studies/Ethiopia.pdf	n.d.
29	19	Ethiopia	*Assessment of the Ethiopian National Health Information System Final Report*	WHO Health Metrics Network	http://www.who.int/healthmetrics/en/	n.d.
30	20	Gambia	*Health Metrics Network The Gambia Health Information System*	WHO Health Metrics Network	http://www.who.int/healthmetrics/en/	2007
31	21	Georgia	*Health Information System Assessment in Georgia using Health Metrics Network Framework and Health Information System Situation Analysis Tool*	WHO Health Metrics Network	http://www.who.int/healthmetrics/en/	2006
32	22	Ghana	*Ghana implementing a national human resources for health plan*	Global Health Workforce Alliance, WHO	http://www.who.int/workforcealliance/knowledge/case_studies/Ghana.pdf	2008
33	22	Ghana	*Ghana Health Information System: Sub-National Health Information System Assessment*	WHO Health Metics Network	http://www.who.int/healthmetrics/en/	2006
34	23	India	*'By papers and pens, you can only do so much': views about accountability and human resource management from Indian government health administrators and workers*	Asha, George	*Int J Health Plann Manage* 2009; 24:205-224.	2009
35	23	India	*Not Enough Here....Too many there...Health Workforce in India (WHO)*	World Health Organization Country Office for India	http://www.whoindia.org/LinkFiles/Human_Resources_Health_Workforce_in_India_-_Apr07.pdf	2007
36	23	India	*Health Human Resources*	Government of India. Central Bureau of Health Intelligence. Ministry of Health and Family Welfare	*National Health Profile (NHP) of India*; http://cbhidghs.nic.in/writereaddata/linkimages/Health%20Human%20Resources4484269844.pdf	2007
37	23	India	*India's Health Workforce: Size, Composition, and Distribution.*	Rao K, Bhatnagar A, Berman, P et al. Public Health Foundation of India, World Bank	http://phfirc.org/hrhindia.org/assets/images/Paper-I.pdf	2008
38	24	Indonesia	*Indonesia Health Information System Review and Assessment*	WHO Health Metrics Network	http://www.who.int/healthmetrics/en/	2007
39	25	Ireland	*Nursing Human Resources in Ireland: Case Study*	Buchan J, International Centre for Human Resources in Nursing	http://www.ichrn.com/publications/casestudies/Ireland_CaseStudy_aug-11-08-EN.pdf	2009
40	26	Kenya	*Developing a nursing database system in Kenya*	Riley PL, Vindigni SM, Arudo J, et al.	*Health Serv Res* 2007, 42:1389-1405. http://www.ncbi.nlm.nih.gov/pmc/articles/PMC1955370/pdf/hesr0042-1389.pdf	2007
41	26	Kenya	*Report for the Assessment of the Health Information System of Kenya*	WHO Health Metics Network	http://www.who.int/healthmetrics/en/	2008
42	26	Kenya	*Rising to the Challenges of Human Resources for Health in Kenya: Developing Empirical Evidence for Policy Making*	Chankova S, Kombe G, Muchiri S, et al. Abt Associates, USAID	HRH Global Resource Center http://www.hrhresourcecenter.org/node/592	2006
43	26	Kenya	*HR mapping of the health sector in Kenya: the foundation for effective HR management*	James J, Muchiri S, Kenya Ministry of Health	HLSP Institute and Ministry of Health, Kenya. Human Resources for Health Global Resource Center. http://www.hrhresourcecenter.org/node/869	2006
44	27	Kyrgyz	*Kyrgyz Republic Health Information system: Review and assessment*	WHO Health Metrics Network	http://www.who.int/healthmetrics/en/	2007
45	28	Laos	*Lao Health Information System: Review and Assessment*	WHO Health Metrics Network	http://www.who.int/healthmetrics/en/	2006
46	29	Lesotho	*Health Metrics Network Lesotho Health Information System Review and Assessment*	WHO Health Metics Network	http://www.who.int/healthmetrics/en/	2007
47	30	Malawi	*Human Resources - Capacity Development within the Health Sector Needs Assessment Study*	Republic of Malawi, Ministry of Health, Malawi Health SWAp Donor Group, GTZ	http://www.scotland-malawipartnership.org/documents/37-Malawi_Health_SWAp-Needs_Assessment_GTZ_Final_Report.pdf	2007
48	30	Malawi	*Human Resources and Financing for the Health Sector in Malawi*	Africa Human Development, Africa Region, World Bank, Ministry of Health Malawi	HRH Global Resource Center, http://www.hrhresourcecenter.org/node/2563	2010
49	30	Malawi	*Malawi Human Resources Information System*	Schenck-Yglesias C, JHPIEGO	HRH Global Resource Center, http://www.hrhresourcecenter.org/node/706 http://pdf.usaid.gov/pdf_docs/PNACX173.pdf	2004
50	30	Malawi	*Country case study. Malawi's Emergency Human Resources Program*	Global Health Workforce Allicance, WHO	http://www.who.int/workforcealliance/knowledge/case_studies/Malawi.pdf	2008
51	31	Mali	*Rapport d'evaluation du systeme national d'information sanitaire par l'outil du reseau de metrologie sanitaire*	WHO Health Metrics Network	http://www.who.int/healthmetrics/en/	2008
52	32	Mauritus	*Mauritus Health Information System Development Strategy and Plan*	WHO Health Metics Network	http://www.who.int/healthmetrics/en/	2008

Article no.	Country no.	Country	Title	Author/Publisher	Source	Year
53	33	Mexico	*Salud en las Americas, 2007. Volumen II- Paises.*	SALUD EN LAS AMÉRICAS, 2007.VOLUMEN II–PAÍSES	www.mex.ops-oms.org/documentos/publicaciones/MéxicoSpanish.pdf	2007
54	34	Moldova	*The evaluation of the Health Information System in the Republic of Moldova*	WHO Health Metrics Network	http://www.who.int/healthmetrics/en/	2007
55	35	Mongolia	*Mongolia Health Information System: Assessment Report*	WHO Health Metrics Network	http://www.who.int/healthmetrics/en/	2007
56	36	Mozambique	*Mozambique Ministry of Health - descriptions of SIFo (subsystem of training information) and SIP (subsystem of personnel information)*	Mozambique Ministry of Health	http://www.misau.gov.mz/pt/misau/drh_direccao_de_recursos_humanos	2009
57	37	Myanmar	*Myanmar Health Information System: Review and Assessment*	WHO Health Metrics Network	http://www.who.int/healthmetrics/en/	2007
58	38	Namibia	*Namibia Health Information System Profile and Assessment*	WHO Health Metrics Network	http://www.who.int/healthmetrics/en/	2005
59	39	Nicaragua	*Diagnostico de situacion sistema de informacion en salud*	WHO Health Metics Network	http://www.who.int/healthmetrics/en/	2007
60	40	Nigeria	*Health workforce country profile for Nigeria*	Africa Health Workforce Observatory, WHO	http://www.afro.who.int/hrh-observatory/country_information/countryprofiles/Nigeria_HRH_Country_Profile_2008.pdf	2008
61	40	Nigeria	*A Situation Assessment of Human Resources in the Public Health Sector in Nigeria*	Chankova S, Nguyen H, Chipanta D, et al, Federal Government of Nigeria and Partners for Health ReformPlus, USAID	HRH Global Resource Center, http://www.hrhresourcecenter.org/node/1007	2006
62	41	Oman	*Human resources for health in the Sultanate of Oman*	Ministry of Health, Sultanate of Oman	http://www.emro.who.int/hrh-obs/media/pdf/oma_hrh_08.pdf	2008
63	42	Palestine	*Human resources for health in Palestine: a policy analysis. Part I: Current situation and recent developments.*	Hamdan M, Defever M	*Health Policy* 2003, 64:243-259	2003
64	42	Palestine	*Human resources for health in Palestine: a policy analysis. Part II. The process of policy formulation and implementation.*	Hamdan M, Defever M	*Health Policy* 2003, 64,2:261-273	2003
65	43	Papua New Guinea	*Information systems for health sector monitoring in Papua New Guinea*	Cibulskis RE, Hiawalyer G	*Bull World Health Organ* 2002;80:752-758. http://www.ncbi.nlm.nih.gov/pmc/articles/PMC2567603/pdf/12378295.pdf	2002
66	44	Paraguay	*Situational Study of Human Resources in Paraguay*	Paraguay Ministry of Public Health and Social Welfare	www.observatoriorh.org/esp/docs/datos-de-paraguay.doc	2000
67	45	Philippines	*Philippine Health Information System: Review and Assessment*	WHO Health Metrics Network	http://www.who.int/healthmetrics/en/	2007
68	46	Portugal	*The state of the health workforce in Portugal*	Biscaia, Andre; Martins, Jose; Carrolo Margarida	*Cah. Socio. Demo. Med.* 47: 257-272.	2009
69	47	Rwanda	*Evaluation of the Capacity Project's Human Resource Information Systems Strengthening Process in Swaziland, Uganda, and Rwanda*	de Vries D, Blair G, Morgan Kairie, The Capacity Project, USAID	HRH Global Resource Center http://www.hrhresourcecenter.org/node/2944	2009
70	48	Senegal	*Rapport d'evaluation du systeme d'information sanitaire au Senegal*	WHO Health Metrics Network	http://www.who.int/healthmetrics/en/	2007
71	49	Sierra Leone	*Report on an Assessment of the Sierre Leone Health Information System*	WHO Health Metrics Network	http://www.who.int/healthmetrics/en/	2006
72	50	South Africa	*Assessment of the health information system in South Africa*	WHO Health Metrics Network	http://www.who.int/healthmetrics/en/	2009
73	51	Sudan	*Sudan Health Information System: Review and Assessment*	WHO Health Metrics Network	http://www.who.int/healthmetrics/en/	2007
74	51	Sudan	*Mapping of Human Resources for Health: Final report*	Bushara I, Badr ES, Federal Ministry of Health Sudan	http://www.emro.who.int/sudan/pdf/Mapping%20of%20HRH%20Sudan.pdf	2005
75	52	Suriname	*Assessment of the Suriname Health Information Systems*	Crape B (ed), Suriname Minisitry of Health, PAHO, WHO	www.paho.org/English/DD/AIS/surreport.pdf	2007
76	53	Swaziland	*Swaziland Health Information System: Review and Assessment*	WHO Health Metrics Network	http://www.who.int/healthmetrics/en/	2007
69*	53	Swaziland	*Evaluation of the Capacity Project's Human Resource Information Systems Strengthening Process in Swaziland, Uganda, and Rwanda*	de Vries D, Blair G, Morgan Kairie, The Capacity Project, USAID	HRH Global Resource Center http://www.hrhresourcecenter.org/node/2944	2009
77	54	Tajikistan	*The assessment of the health information system in Tajikistan*	WHO Health Metrics Network	http://www.who.int/healthmetrics/en/	2009
78	55	Tanzania	*Health Sector Performance Profile Report (2009)*	Ministry of Health and Social Welfare	http://hdptz.esealtd.com/fileadmin/documents/Other_Health_Meetings/Health_Sector_Performance_Profile_Report_2009_4_Nov_FINAL_DRAFT.pdf	2007
79	55	Tanzania	*Assessment of the country of health information system in Tanzania*	WHO Health Metrics Network	http://www.who.int/healthmetrics/en/	2007

Article no.	Country no.	Country	Title	Author/Publisher	Source	Year
80	56	Tunisia	*Rapport detaille du projet de renforcement d'information sanitaire en Tunisie*	WHO Health Metrics Network	http://www.who.int/healthmetrics/en/	2006
81	57	Uganda	*Assessment of the Health Information System in Uganda*	WHO Health Metrics Network	http://www.who.int/healthmetrics/en/	2007
69*	57	Uganda	*Evaluation of the Capacity Project's Human Resource Information Systems Strengthening Process in Swaziland, Uganda, and Rwanda*	de Vries D, Blair G, Morgan K, The Capacity Project, USAID	HRH Global Resource Center, http://www.hrhresourcecenter.org/node/2944	2009
82	58	United States of America	*National Center for Health Workforce Analysis: U.S. Health Workforce Personnel Factbook*	Observatory of Human Resources in the Health Sector Reforms, PAHO, WHO	www.observatoriorh.org/eng/basic_data.html	n.d.
83	58	United States of America	*Counting Physicians: Inconsistencies in a Commonly Used Source for Workforce Analysis*	Freed G, Nahra T, Wheeler J; Research Advisory Committee of the American Board of Pediatrics	*Acad Med* 2006, 81:847-852	2006
84	58	United States of America	*Registered Nurse Survey 2004*	Health Resources and Services Administration, Department of Health and Human Resources	ftp://ftp.hrsa.gov/bhpr/workforce/0306rnss.pdf	2004
85	58	United States of America	*Self-Reported Physician Specialties and the Primary Care Content of Medical Practice: A Study of the AMA Physician Masterfile*	Shea J, Kletke PR, Wozniak GD, et al.	*Med Care* 1999, 37: 333-338. http://www.jstor.org/stable/3767442	2009
86	58	United Sates of America	*Shaping HealthWorkforce Policy Through Data-Driven Analyses: The North Carolina Health Professions Data System*	King J, Holmes M	*NC Med J* 2008, 69:139-141. http://www.shepscenter.unc.edu/research_programs/rural/publications/index.html	2008
87	58	United States of America	*HRSA State Health Workforce Data Resource Guide*	Health Resources and Services Administration, Bureau of Health Professions, National Center for Health Workforce Information and Analysis	ftp://ftp.hrsa.gov/bhpr/workforceprofiles/guide.pdf	n.d.
88	58	United States of America	*Public Health Workforce Enumeration*	Gebbie K, Raziano A, Elliott S	*Am J Public Health* 2009, 99: 786-787	2009
89	59	United Kingdom	*Health Sector reform and human resources: lessons from the United Kingdom*	Buchan J	*Health Policy Plan* 2000, 15: 319-325	2000
90	60	Vanuatu	*Vanuatu Health Information System: Review and Assessment*	WHO Health Metrics Network	http://www.who.int/healthmetrics/en/	2007
91	60	Vanuatu	*Mapping HRH Profiles from 15 Pacific Island Countries*	University of New South Wales, School of Public Health and Community Medicine.	HRH Global Resource Center http://www.hrhresourcecenter.org/node/3187	2009
92	61	Vietnam	*Viet Nam Health Information System Review and Assessment*	WHO Health Metrics Network	http://www.who.int/healthmetrics/en/	2006
93	62	Yemen	*Yemen Health Information System: Review and Asessment Report*	WHO Health Metrics Network	http://www.who.int/healthmetrics/en/	2007
94	63	Zambia	*Assessment of the Health Information System in Zambia*	WHO Health Metrics Network	http://www.who.int/healthmetrics/en/	2007
12*		Mercosur region	*Brazil Ministry of Health - Report on Mercosur for Health Work (Annex I : data collection form for health worker database)*	Brazil Ministry of Health	http://portal2.saude.gov.br/portal/arquivos/pdf/cartmercosulespanhol.pdf	2006
91*		Asia-Pacific region	*Mapping HRH Profiles from 15 Pacific Island Countries*	University of New South Wales, School of Public Health and Community Medicine	Human Resources for Health Knowledge Hub, HRH Global Resource Center; http://www.hrhresourcecenter.org/node/3187	2009
95		Southeast Asia / Western Pacific regions	*WHO Minimum HRH data set*	World Health Organization South-East Asia Region and Western Pacific Region. Sydney University of Technology	World Health Organization. http://www.who.int/hrh/documents/hrh_minimum_data_set.pdf	2008
